# Virus-Mediated shRNA Knockdown of Prodynorphin in the Rat Nucleus Accumbens Attenuates Depression-Like Behavior and Cocaine Locomotor Sensitization

**DOI:** 10.1371/journal.pone.0097216

**Published:** 2014-05-09

**Authors:** Ami Cohen, Timothy W. Whitfield, Max Kreifeldt, Pascale Koebel, Brigitte L. Kieffer, Candice Contet, Olivier George, George F. Koob

**Affiliations:** 1 Committee on the Neurobiology of Addictive Disorders, The Scripps Research Institute, La Jolla, California, United States of America; 2 Institut de Génétique et de Biologie Moléculaire et Cellulaire, Translational Medicine and Neurogenetic Programme, UdS Université de Strasbourg, INSERM U964, CNRS UMR7104, Illkirch, France; INSERM/CNRS, France

## Abstract

Dynorphins, endogenous opioid peptides that arise from the precursor protein prodynorphin (*Pdyn*), are hypothesized to be involved in the regulation of mood states and the neuroplasticity associated with addiction. The current study tested the hypothesis that dynorphin in the nucleus accumbens (NAcc) mediates such effects. More specifically, we examined whether knockdown of *Pdyn* within the NAcc in rats would alter the expression of depressive-like and anxiety-like behavior, as well as cocaine locomotor sensitization. Wistar rats were injected with adeno-associated viral (AAV) vectors encoding either a *Pdyn*-specific short hairpin RNA (AAV-shPdyn) or a scrambled shRNA (AAV-shScr) as control. Four weeks later, rats were tested for anxiety-like behavior in the elevated plus maze test and depressive-like behavior in the forced swim test (FST). Finally, rats received one daily injection of saline or cocaine (20 mg/kg, i.p.), followed by assessment of locomotion for 4 consecutive days. Following 3 days of abstinence, the rats completed 2 additional daily cocaine/saline locomotor trials. *Pdyn* knockdown in the NAcc led to a significant reduction in depressive-like behavior in the FST, but had no effect on anxiety-like behavior in the elevated plus maze. *Pdyn* knockdown did not alter baseline locomotor behavior, the locomotor response to acute cocaine, or the initial sensitization of the locomotor response to cocaine over the first 4 cocaine treatment days. However, following 3 days abstinence the locomotor response to the cocaine challenge returned to their original levels in the AAV-shPdyn rats while remaining heightened in the AAV-shScr rats. These results suggest that dynorphin in a very specific area of the nucleus accumbens contributes to depressive-like states and may be involved in neuroadaptations in the NAcc that contribute to the development of cocaine addiction as a persistent and lasting condition.

## Introduction

Dynorphins, a class of opioid peptides that arise from the precursor protein prodynorphin (encoded by the *Pdyn* gene), have been implicated in emotional control and stress responses in general [1], and in the negative emotional states associated with drug addiction in particular [2]. Dynorphins are the primary endogenous ligands of kappa opioid receptors (KOR) and systemic or central KOR activation produces dysphoria in humans [3], [4] and depressive-like behaviors in rodents, including increased elevated reward thresholds in the intracranial self-stimulation test (ICSS) [5], and increased immobilization in the forced swim test (FST) [6]. Conversely, KOR antagonism produces antidepressant-like effects in the FST and ICSS test in rodents [7]–[9]. Moreover, *Pdyn* knockout (KO) mice have shown reduced stress-induced depressive-like behavior in the FST [8]. The dynorphin/KOR system appears to also be involved in anxiety, as systemic administration of KOR antagonists to rodents dose-dependently reduces anxiety-like behavior in the elevated plus maze [10], [11], the open field [11], and the fear-potentiated startle paradigm [10]. In contrast, studies making use of *Pdyn* KO mice showed mixed results, with some studies demonstrating anxiolytic-like behavior in the elevated plus maze, and open field test [12], while others showed increased anxiety-like behavior [13].

Although dynorphin and KORs are abundant throughout the CNS [14], [15], the depressive-like and dysphoric-like effects of dynorphin in general, and in response to stress in particular, have been hypothesized to be mediated by interactions with dopaminergic and serotonergic systems [16], [17]. Dopaminergic cell bodies are located in the ventral tegmental area (VTA) and project to the prefrontal cortex (PFC; the mesocortical system) and to the nucleus accumbens (NAcc, the mesolimbic system). Dopamine secretion, particularly within the NAcc, is known to be important for motivation, reward and mood regulation [18], [19]. Administration of the dynorphin A analog E-2078 into the VTA, NAcc or PFC, induces conditioned place aversion in rats [20], and KOR activation attenuates dopamine release in the NAcc [21], [22], as well as the mPFC [23]. Indeed, KORs on dopaminergic terminals in the PFC mediate the aversive impact of systemic KOR activation [23], and administration of the KOR agonist U50,488 directly into the NAcc shell increases the reward thresholds in the ICSS test [24]. Conversely, intra-NAcc delivery of the KOR antagonist nor-binaltorphimine (norBNI) produces antidepressant-like effect in rodent models of learned helplessness [25], [26]. Thus, KOR-dependent inhibition of dopamine release in the NAcc likely plays a central role in dynorphin-mediated dysphoria [18], [27]. In addition, data suggest that serotonergic projections from the dorsal raphe nucleus (DRN) to the NAcc have a central role in the regulation of aversive behavioral responses by the dynorphin/KOR activation [17]. Specifically, KOR activation in the NAcc induces local increase in serotonin transport, particularly in the synaptic terminals of DRN neurons, and this neuropharmacological action has been hypothesized to contribute to a hyposerotonergic state that may contribute to prodepressive effects [28]. Finally, KOR agonists decrease GABA and glutamate levels in the NAcc [29] and may thus also contribute to the overall reward state regulation via dynorphin/KOR activation. In contrast, the contribution of dynorphin/KOR activation in the NAcc to anxiety-like behavior remains unknown and studies point to the basolateral amygdala as a region in which KOR activation mediates anxiety-like behavior [30], [31].

The inhibitory effects of dynorphin on dopamine and serotonin in the NAcc are consistent with growing evidence for the involvement of the dynorphin/KOR system in drug abuse and addiction. Specifically, the dynorphin/KOR system has been strongly implicated in the negative affective states that follow drug withdrawal and may sustain drug dependence via a process of negative reinforcement [32]–[34]. Moreover, dynorphin may be involved in the process of “sensitization”, long-lasting behavioral and physiological changes that occur as a result of repeated exposure to psychostimulants (particularly cocaine), and that are evident in rodents by an increased locomotor response to a cocaine challenge [35]. The sensitization to the locomotor-enhancing effects of cocaine largely results from increased dopamineergic activity, principally in the NAcc [36]. Co-administration of synthetic KOR agonists and dynorphin with cocaine prevents the sensitization to the locomotor stimulatory effect of cocaine [37]–[39]. However, the delivery of KOR agonists at high concentrations does not well represent the contribution of physiological levels of dynorphin. Indeed, a recent study has shown that the long-lasting KOR antagonist norBNI prevented the sensitization of the motor response to cocaine [40].

As the specificity of dynorphin to KOR is high but not absolute [41], a better strategy for exploring the role of NAcc dynorphin in cocaine sensitization, anxiety and depressive-like behavior, is the use of genetic approaches. Constitutive deletion of the *Pdyn* gene led to mixed results, with one study demonstrating reduced ability of cocaine to increase NAcc dopamine levels and decreased cocaine-evoked locomotor activity in *Pdyn* KO mice [42], and another study demonstrating enhanced cocaine locomotor sensitization with no difference in the acute effects of cocaine in *Pdyn* KO mice [43]. Moreover, Van't Veer *et al.* [44] demonstrated that while constitutive KOR KO mice did not differ from their wild-type counterparts in anxiety-like behavior and sensitization to the locomotor-enhancing effects of cocaine, conditional KO mice lacking KORs in DA-containing neurons showed decreased anxiety-like behavior and potentiated sensitization. Thus, the differences between the KO studies may result, at least partially, from various developmental compensations that complicate interpretation of results in constitutive gene KO studies. Accordingly, KORs were shown to be up-regulated in *Pdyn* KO mice [45]. Furthermore, the KO manipulation in these experiments was global and conclusions about the specific role of the NAcc could not be reached.

In the current study we used a genetic approach of greater temporal and spatial resolution: site-specific *Pdyn* knockdown of adult rats using recombinant adeno-associated viral (rAAV) vectors. This model bypasses the problem of possible developmental compensations for the reduction in prodynorphin production and allows for isolation of the NAcc as the site of knockdown. We injected rAAV vectors expressing a shRNA sequence targeted at the *Pdyn* transcript (shPdyn) or a control scrambled shRNA (shScr) into the NAcc of adult rats and subsequently conducted behavioral assessments of depressive-like behavior, anxiety-like behavior and sensitization to the locomotor enhancing effects of cocaine.

## Materials and Methods

### Animals

Twenty-two male Wistar rats (250–275 g; Charles River, Hollister, CA) were group-housed and maintained on a 12 h/12 h light/dark cycle with *ad libitum* access to food and water, with all behavioral procedures conducted during the dark phase of the cycle. All of the animal procedures were conducted in accordance with the recommendations of the National Institute of Health and approved by the Scripps Research Institute Institutional Animal Care and Use Committee (Permit Number: 08-0015). All surgical procedures were performed under isoflurane anesthesia and all necessary steps were taken to minimize suffering of the animals.

### Viral vectors

Recombinant adeno-associated viral (rAAV) vectors were produced using an AAV2 helper-free system (Agilent Technologies), as described in Darcq et al. [46]. The rAAV vectors expressed a shRNA sequence under the control of mU6 promoter, along with eGFP under the control of the CMV promoter. The shRNA sequence targeted at the *Pdyn* transcript (shPdyn, 5′-GGTTGCTTTGGAAGAAGGCTACA-3′) was selected using BLOCK-iT RNAi designer (Life Technologies, Grand Island, NY) and reduced expression of a rat prodynorphin-eGFP fusion in transfected COS-1 cells by 98% compared to a scramble shRNA sequence with no homology to any transcript (shScr, 5′-GCGCTTAGCTGTAGGATTC-3′), as analyzed by fluorescence-activated cell sorting (data not shown).

### Intracranial viral infusions

Rats were anesthetized (1–3% isoflurane in oxygen mixture) and placed in a Kopf stereotaxic instrument. Double cannulae (gauge; Plastics One, Roanoke, VA) were inserted bilaterally above the left and right NAcc (coordinates relative to bregma: anterior/posterior, −1.7 mm; medial/lateral, ±1.5 mm; dorsal/ventral, −5.8 mm from skull surface) [47[. 2 µl (approximately 5.10*8 physical particles, measured by QPCR using a linearized standard plasmid) of either the rAAV-shPdyn (N = 12) or the rAAV-shScr (N = 10) were infused at a rate of 0.25 µl/min using injectors protruding 2 mm beyond the cannula. Upon completion of the injection the injectors were left in place for 10 additional minutes to assure adequate diffusion of the solution. Infusions were delivered via polyethylene tubing (PE 20) that was connected to a Hamilton 10 µl syringe.

### Experimental design

**Figure 1:**
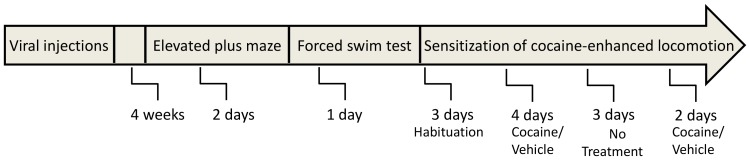
Experimental design. Rats were administered with either rAAV-shPdyn (N = 12) or the control rAAV-shScr (N = 10) into their nucleus accumbens (NAcc). 4 weeks later, rats were tested for anxiety-like behavior in the elevated plus maze and then subjected to the forced swim test (FST), a measure of depressive-like behavior, for 2 consecutive days. Subsequently, the rats were habituated to the locomotor activity apparatus for 1 hour per day, for 3 consecutive days. On the subsequent 4 test days rats were placed in the activity cages for 1 hour recording of baseline locomotor activity and then half were injected i.p with cocaine (20 mg/kg/1 ml saline) and half with saline before being placed back in the activity apparatus cages for 1 additional hour to record post-treatment behavior. The rats were subjected to 4 consecutive test days, with each rat receiving the same drug treatment each day, followed by 3 days of rest and 2 additional test days. Subsequently rats were sacrificed and their brains extracted.

Behavioral testing began four weeks following the viral-vector injections, including the elevated plus maze, the forced swim test and cocaine-induced locomotor sensitization, according to the timeline shown in Fig 1.

### Elevated plus maze

The elevated plus maze consisted of four arms (50 cm length×10 cm width) elevated 100 cm above the floor. Two of the arms had 40 cm high dark walls (closed arms), and two had 0.5 cm high ledges (open arms). The arms were angled at 90° to each other. The apparatus was placed in a quiet room dimmed to provide 10–20 lux of illumination on the open arms and <0.5 lux within the closed arms. The rats were placed in the center of the maze facing a closed arm and removed after 5 min. The apparatus was wiped with water and dried between tests.

### Forced swim test

The forced swim test (FST) was composed of a 2-day procedure [48], [49]. On the first day (pretest), rats were individually placed in two clear Plexiglas cylinders (25 cm diameter ×63 cm height) that were filled with water (22–24°C, 45 cm depth) for 10 min. The cylinders were separated from one another by a dark screen. The water was replaced between subjects. On the second day (test), the rats were placed in the same condition for 5 min. After each session, rats were towel dried and returned to their home cage. Swimming behaviors were videotaped throughout the session. The dependent measures included immobility (representing “depression-like behavior”), characterized by floating with movements merely essential to keep the nose above the water, swimming, which is characterized by horizontal movements across the water surface, and climbing, which is characterized by vertical movements against walls. These behaviors of the rats were scored using a time-sampling method in which swimming, climbing or immobility was rated at a 5 second intervals [48], [50].

### Cocaine sensitization of locomotor activity

The locomotor activity apparatus consisted of 16 wire mesh cages (20×25×36 cm) equipped with 2 horizontal infrared photobeams situated along the long axis of the cage, 2 cm above the floor and 16 cm between themselves. During testing, white noise (70 dB) was present and photocell beam break counts were recorded, with photocell counts indicative of movement and cage crossing of movement from one side of the cage to another. Rats were habituated to the cages for 1 hour per day, for 3 consecutive days. On the subsequent test days rats were placed in the activity cages for 1 hour for habituation and recording of baseline locomotor activity and then half were injected i.p with cocaine (20 mg/kg/1 ml saline) and half with saline before being placed back in the activity apparatus cages for 1 additional hour to record post-treatment behavior. The rats were subjected to 4 consecutive test days, with each rat receiving the same drug treatment each day, followed by 3 days of rest and 2 additional test days, to examine the lasting effects of the repeated cocaine administration (Fig 1).

### In situ hybridization

After completion of all behavioral tests rats were anesthetized using isoflurane and decapitated. Brains were immediately harvested, snap-frozen in isopentane and stored at −80°C. Six series of 20-µm coronal cryostat sections were collected on Superfrost Plus slides. A first series was processed for *in situ* hybridization with a GFP probe to assess location and extent of viral transduction. A second series was processed for *in situ* hybridization with a *Pdyn* probe to evaluate silencing efficiency within the transduced area. A plasmid containing the EGFP sequence subcloned into pGEM-T Easy was donated by Dr. Richard Rivera (The Scripps Research Institute, La Jolla, CA). A plasmid containing a 1.7-kb fragment of the rat *Pdyn* gene subcloned into pSP64 was obtained from Dr. Joel Elmquist (UT Southwestern Medical Center, Dallas, TX). Digoxigenin (DIG)-labeled riboprobes were synthesized using a kit (Roche, Indianapolis, IN). Sections were post-fixed in paraformaldehyde (PFA) 4%, and then acetylated in 0.1 M triethanolamine pH 8.0, acetic acid 0.2%. Following washes in salt sodium citrate (SSC) 2x, sections were dehydrated and defatted in a graded ethanol/chloroform series. Pre-hybridization and hybridization were performed at 70°C in a buffer containing 50% formamide, SSC 2x, Ficoll 0.1%, polyvinylpyrrolidone 0.1%, bovine serum albumin (BSA) 0.1%, sheared salmon sperm DNA (0.5 mg/mL) and yeast RNA (0.25 mg/mL). Probes were diluted in the hybridization buffer (800 ng/mL) and incubated overnight on slides. Post-hybridization washes were performed in 50% formamide, SSC 2x, Tween-20 0.1%. Sections were then blocked for 1 h and incubated with anti-DIG antibody overnight at 4°C (Roche, 1∶2000) in MABT buffer (0.1 M maleic acid pH 7.5, 0.15 M NaCl, Tween-20 0.1%) containing 10% normal goat serum (NGS). Following washes in MABT and incubation in detection buffer (0.1 M Tris-HCl pH 9.5, 0.1 M NaCl, 0.05 M MgCl_2_, Tween-20 0.1%), the reaction with NBT-BCIP was allowed to develop in the dark for 24 h at room temperature. Slides were rinsed, air dried and mounted in DPX (Sigma, St-Louis, MO). Sections were photographed using a Zeiss Axiophot microscope equipped with a QImaging Retiga 2000R color digital camera and QCapture software at 5x magnification. The anterior commissure was included in each picture as an anatomical landmark to enable overlay. Optical density of the hybridization signal was analyzed using NIH Image J software, with each section corrected for background, and expressed as arbitrary units.

To estimate the transduction volume (V), the shape of the transduced region was approximated to an ellipsoid. For each hemisphere of each rat, the section showing the widest area of GFP expression was used to measure the area of the cross section passing through the center of the ellipsoid (A). The GFP signal was then tracked across coronal sections to estimate the anteroposterior extent of transduction based on section thickness (z). The transduction volume was computed using the following formula: V = 2/3*A*z. To calculate silencing efficiency, the section showing the widest area of GFP expression was overlaid onto the corresponding serial section processed for *Pdyn in situ* hybridization. The optical density of *Pdyn* signal within the area of GFP expression was divided by the measured area to obtain the density per 1 mm^3^. These results were then normalized and expressed as a ratio of control (shScr) levels. For statistical analysis, values obtained from each hemisphere of each rat were considered as independent entries.

### Data analysis

Results of the FST, elevated plus maze and *Pdyn* densities were analyzed using *t*-test comparisons. Beam breaks in the locomotor tests were analyzed using area under the curve computation that was subjected to repeated-measures analysis of variance (ANOVA) or to one-way ANOVA on single test days. When applicable, AVOVA was followed by the Newman-Keuls multiple comparison *post hoc* test. In all cases, a normality test and equal variance test were performed before the ANOVA to ensure its validity.

## Results

**Figure 2:**
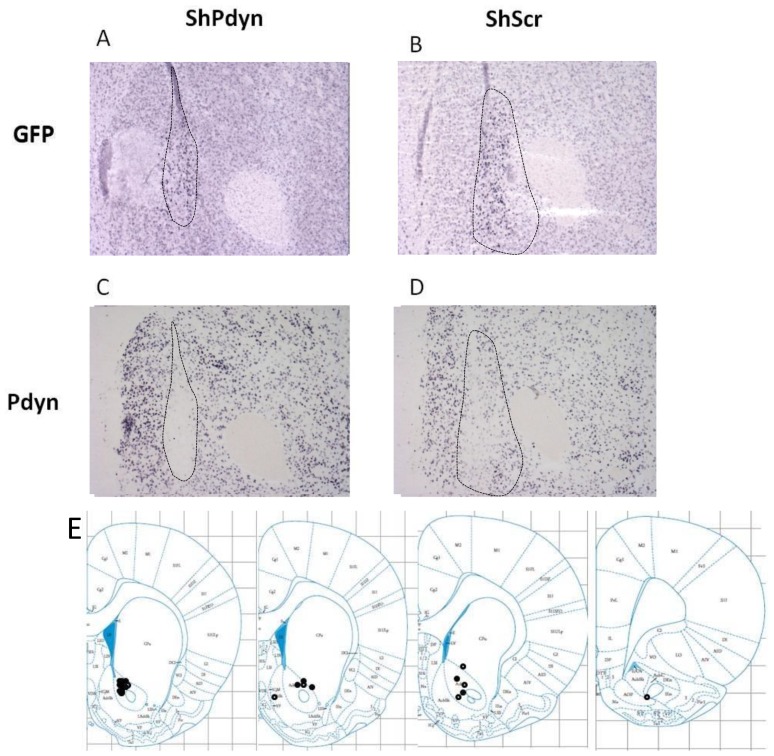
*In situ* hybridization results. *In situ* hybridization with a GFP probe was conducted to assess location and extent of viral transduction in rats administered with a rAAV viral vector expressing a shRNA sequence directed at *Pdyn* transcript(shPdyn) or a control scramble sequence (shScr) into their nucleus accumbens (NAcc). Figures A (shPdyn rat) and B (shScr rat) portray the typical limited transduction volume within the NAcc observed in all subjects. *In situ* hybridization with a *Pdyn* probe demonstrated substantial reduction of *Pdyn* mRNA expression in the transduced region of the shPdyn rats (C) compared to the shScr rats (D). The locations of the rAAV injection sites in each rat are depicted in panel E. Solid black circles: shPdyn rats; white circles: shScr rats.

**Figure 3:**
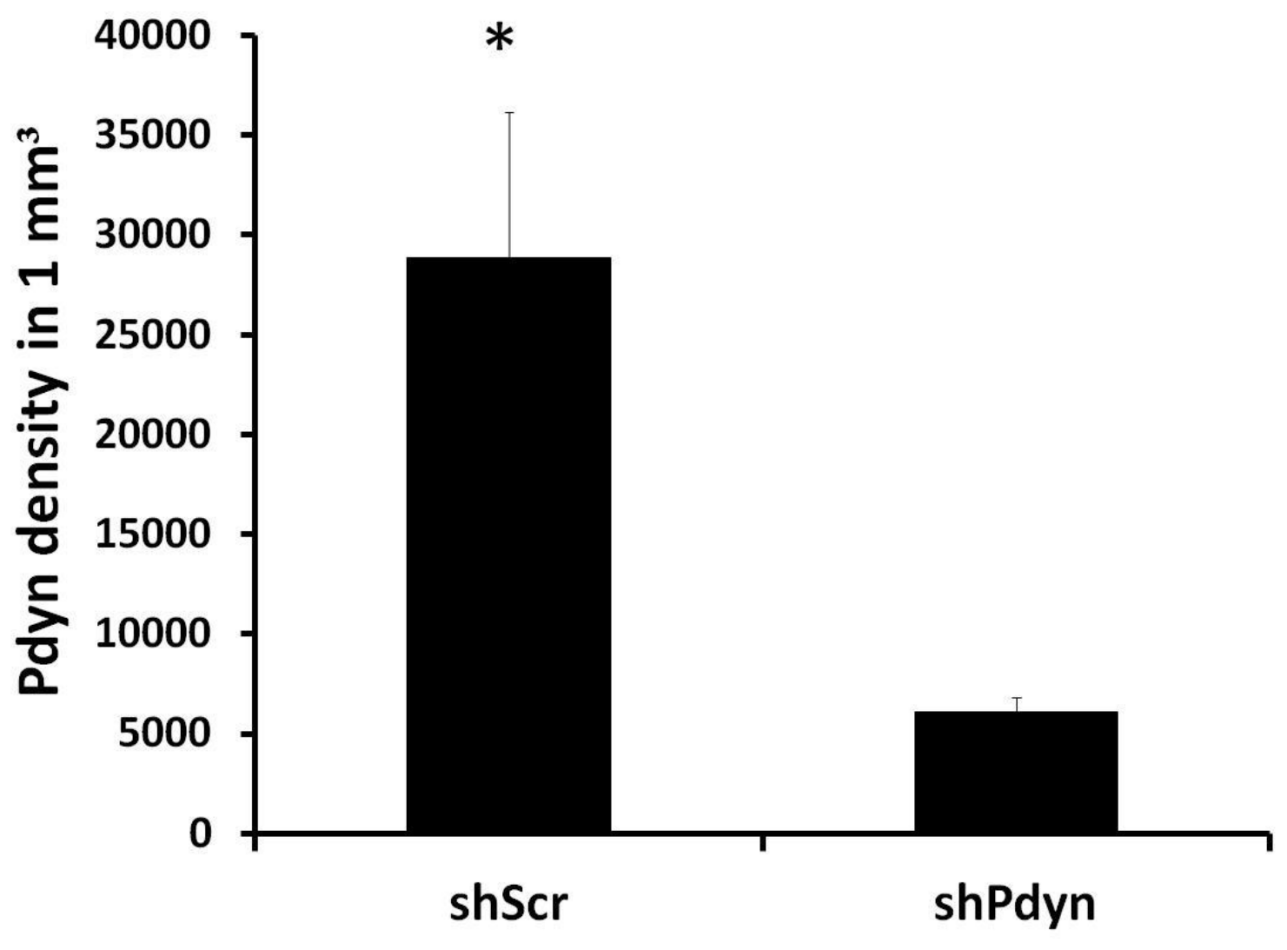
Efficacy of prodynorphin (*Pdyn*) knockdown procedure. The optical density of *Pdyn* signal within the area of GFP expression was divided by the measured area to obtain the density per 1 mm^3^. The viral vector expressing a shRNA sequence directed at *Pdyn* transcript (shPdyn) led to a significant reduction of *Pdyn* mRNA expression in the transduced region compared to the control shScr vector. N = 5–6, *p<0.001.

Injection of the rAAV-shRNA vectors into the NAcc produced a limited transduction volume, as reflected by the density of GFP (Fig. 2A–E). The volume of the transduced region was 1.12±0.094 mm^3^. The rAAV-shPdyn vector led to a significant knockdown of *Pdyn* mRNA expression in the transduced region compared to the rAAV-shScr vector (79% reduction; t34 = 3.92; *P*<0.001; Fig 3).

**Figure 4:**
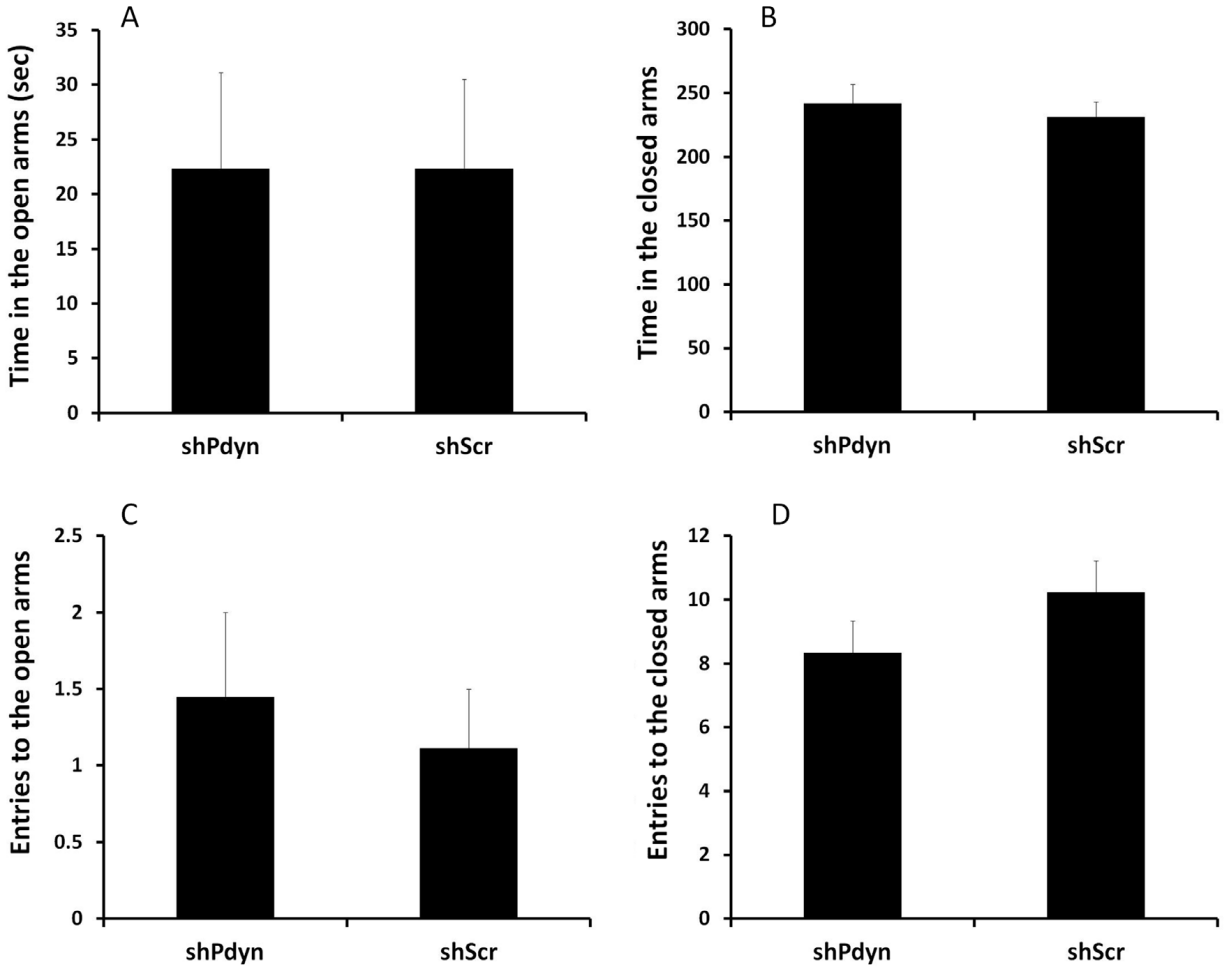
Prodynorphin (*Pdyn*) knockdown in the NAcc does not alter anxiety-like behavior. A) Time in the open arms of the elevated plus maze, a measure of anxiety-like behavior. B) Time in the closed arms of the elevated plus maze. The Groups did not differ in the number of entries into the open arms (C) or the closed arms (D) of the elevated plus maze, suggesting that the results were not affected by differences in locomotor activity. N = 10.

**Figure 5:**
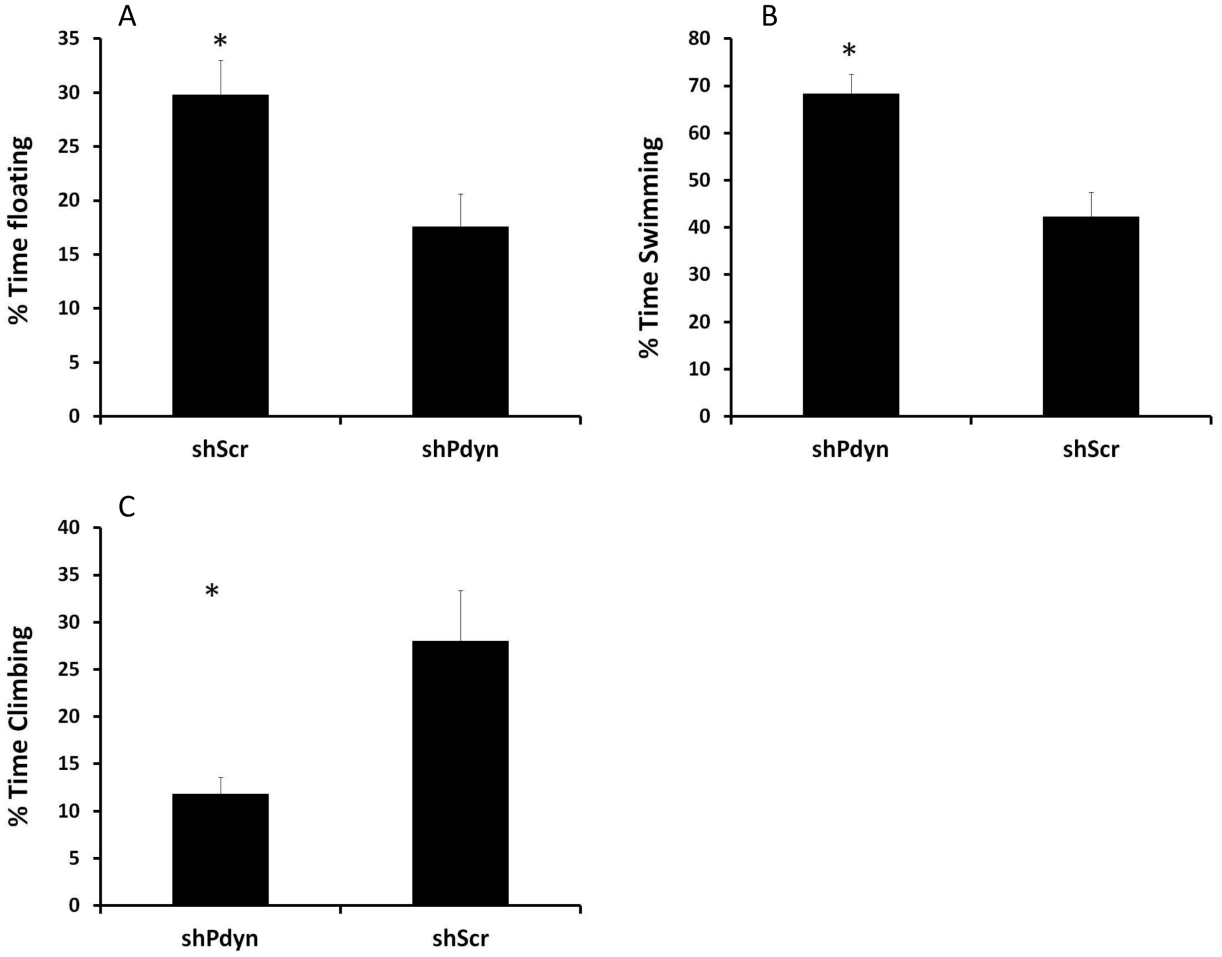
Prodynorphin (*Pdyn*) knockdown in the NAcc attenuated depression-like behavior. A) % of the time in the forced swim test spent floating, a measure of depressive-like behavior. B) % of the time in the forced swim test spent swimming. C) % of the time in the forced swim test spent climbing. N = 10, *p<0.01.

Anxiety-like behavior was similar between shPdyn and shScr rats, reflected by a similar amount of time spent in the open arms of the elevated plus maze (*P*>0.05; Fig 4). Note that the groups did not differ also in the time spent in the closed arm entries (*P*>0.05) or open arm entries (*P*>0.05), suggesting that the results were not affected by differences in locomotion. However, shPdyn rats demonstrated reduced depression-like behavior in the forced swim test (FST), as reflected by lower percent of time spent floating (t19 = 2.74, *P*<0.01; Fig 5A), and increased percent of time spent swimming (t19 = 3.94, *P*<0.01; Fig 5B). Interestingly, climbing behavior was reduced in the shPdyn rats (t19 = 3.07, *P*<0.01; Fig 5C). Notably, on the forced-swim pretest, the groups did not differ in the % of time spent swimming (shPdyn: 76.41±2.99%, shScr: 73.16±4.68%, *P*>0.05), floating (shPdyn: 6.37±1.49%, shScr: 8.4±2.96%, *P*>0.05) or climbing (shPdyn: 14.4±1.95%, shScr: 16.39±4.27%, *P*>0.05).

**Figure 6:**
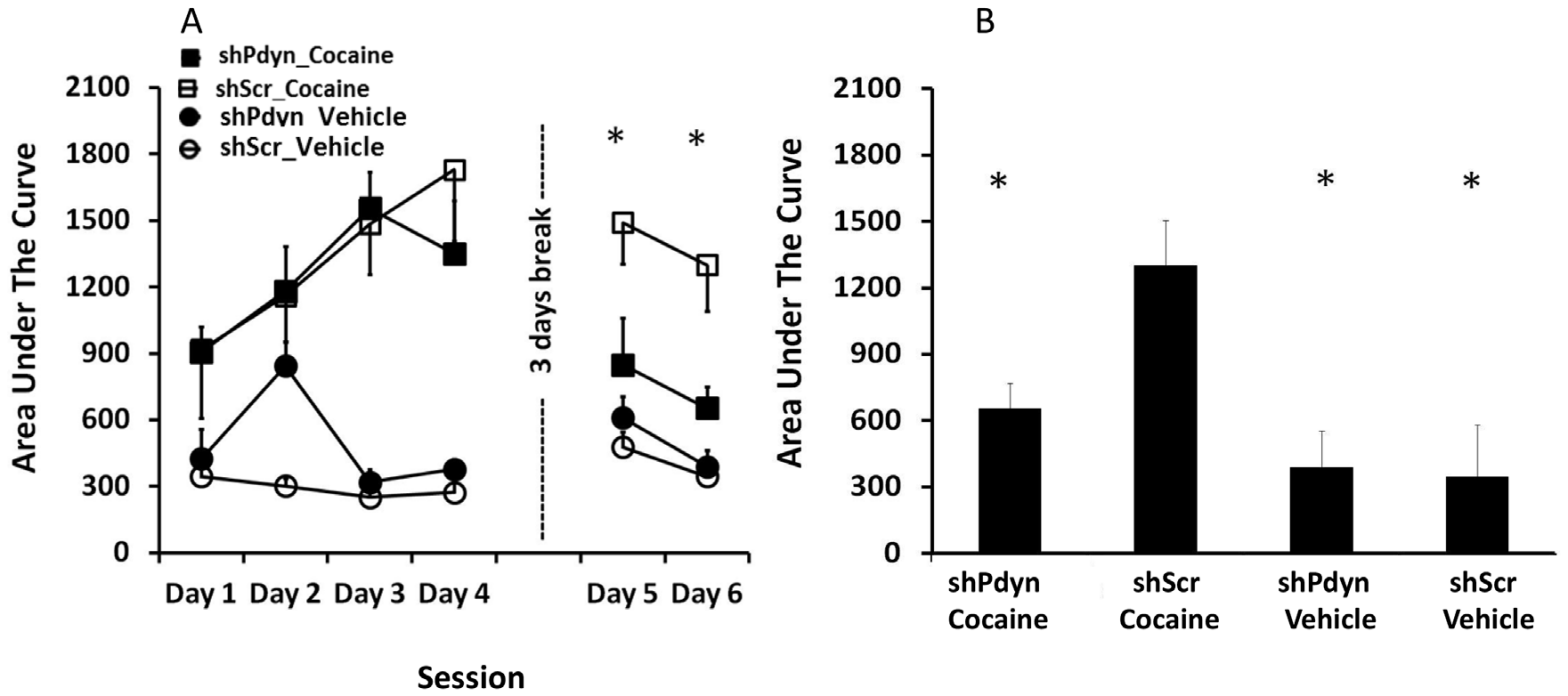
Prodynorphin (*Pdyn*) knockdown in the NAcc attenuated cocaine sensitization. Beam breaks in the locomotor chambers were transformed to area under the curve (AUC). A) shPdyn and shScr rats showed similar level of sensitization in their locomotor response to cocaine during the first 4 days of treatment, but shPdyn rats demonstrated a significant reduction in their cocaine-induced locomotion when tested 72 h later. B) There was a significant difference between the AUC data of the study groups on the last 2 test days, with AUC scores of the cocaine-treated shPdyn rats significantly lower than that of the cocaine-treated shScr rats and not different from the saline-treated shPdyn rats. N = 5–6, *p<0.05.

A two way ANOVA on the locomotor response to acute cocaine/vehicle injection (Fig 6A: session 1) revealed a main effect of Cocaine (f(1,18) = 9.487, *P*<0.01), and no main effect of Vector (shPdyn vs, shScr, f(1,18) = 0.041, *P*>0.05) or a Cocaine × Vector interaction (f(1,18) = 0.069, *P*>0.05) suggesting that shPdyn and shScr rats did not differ in their response to acute cocaine or in their locomotion in response to saline. However, shPdyn and shScr rats did differ in their response to repeated cocaine injections. In particular, while shPdyn and shScr rats showed similar level of sensitization in their locomotor response to cocaine during the first 4 days of treatment, shPdyn rats demonstrated a significant reduction in their cocaine-induced locomotion when tested 72h later (Fig 6A). Specifically, repeated-measures ANOVA revealed a significant Time × Group interaction (f(15,90) = 2.646, *P*<0.01). Accordingly, a one way ANOVA revealed a significant difference between the AUC data of the study groups on the last 2 test days (day 5: f(3,18) = 7.64, *P*<0.01; day 6: f(3,18) = 13.41, *P*<0.01; Fig 6B). Subsequent Newman-Keuls post-hoc tests verified that on both days AUC scores of the cocaine-treated shPdyn rats were significantly lower than that of the cocaine-treated shScr rats (*p*<0.05), and not different from the saline-treated shPdyn rats (*P*>0.05).

## Discussion

This study was designed to examine the role of dynorphin in the nucleus accumbens (NAcc) in functions related to mood regulation and the stimulatory effects of cocaine, as it has been suggested that dynorphin mediates negative mood states (e.g. depression and anxiety) and the dependence-related effects of repeated exposure to cocaine [1], [2]. The current study demonstrated that knockdown of prodynorphin (*Pdyn*) in the NAcc using a shRNA-encoding AAV vector has no effect on anxiety-like behavior in the elevated plus maze but reduces locomotor sensitization following repeated cocaine administration and leads to significant reduction in depressive-like behavior in the forced swim test (FST). In the FST, rats are exposed to water and, following initial engagement in active escape-related behaviors (swimming and climbing), demonstrate passive immobile behavior, which is believed to reflect “behavioral despair” [51]. This procedure has been criticized as having poor face and construct validities but has impressive predictive validity, with immobility decreasing in response to a wide range of antidepressant treatments and increasing in response to various stressors [52]. One major concern with the FST is that manipulations that alter motor activity may give “false positive/negative” effects [51], [52]. However, in the current study no differences between the groups in basal locomotor behavior have been observed in the elevated plus maze or the locomotor chambers, suggesting that the effects observed in the FST were depressive-like rather than decreased locomotor activity.

These findings greatly add to our understanding of the role of dynorphin expression in the NAcc in regulating mood states and cocaine locomotor sensitization due to the particular advantages of the viral vector-mediated knockdown method over more traditional pharmacological and genetic procedures that were commonly used to alter the dynorphin/KOR system. First, KOR agonists are usually administered acutely in doses which may be much higher than the relevant physiological levels, and that do not represent chronic elevation in release. Second, although dynorphin-KOR specificity is high, it is not absolute and dynorphins can bind, for example, also to mu opioid receptors, depending on the specific dynorphin [41], [53]. In addition, the use of traditional constitutive KO models is usually limited to mice, lacks anatomic specificity and can be difficult to interpret due to compensatory developmental changes that may occur in response to the defective gene.

The NAcc, known to be important for motivation, reward and addiction, appears to be central for mediating many of the behavioral effects of dynorphin/KOR activation. KORs are abundant on dopamine terminals in the NAcc [27], and KOR activation attenuates dopamine release in the NAcc [21], [22], an effect that is consistent with hypohedonic-like states [54]. Indeed, intra-NAcc delivery of KOR agonists increased the reward thresholds in the ICSS test, which is a common animal model of decreased reward [24], while intra-NAcc delivery of KOR antagonists induce antidepressive-like effects in the learned helplessness paradigm [25], [26]. Altogether, there is considerable evidence supporting the role of NAcc dynorphin activation in mood regulation. The current study substantially adds to these findings by demonstrating that attenuating dynorphin expression (rather than KOR activity) in the NAcc substantially diminishes depressive-like behavior (immobility) in the FST. It is interesting to note that while shPdyn rats showed reduced immobility and increased swimming, consistent with antidepressant effect, their climbing behavior was also reduced. A pattern of increased swimming, with no change, or even reduction, in climbing is typical to antidepressants that impact the serotonergic system [55], [56]. This is consistent with reports that suggest that serotonergic projections from the dorsal raphe nucleus (DRN) to the NAcc have a central role in the regulation of aversive behavioral responses by dynorphin/KOR activation [17]. KOR activation in the NAcc induces local increase in serotonin transport, and this could contribute to a hyposerotonergic state that may contribute to prodepressive effects [28]. Thus, the results of the current study may, at least partially, reflect prevention of this hyposerotenergic state.

It is important to note that our results by themselves do not necessarily mean that dynorphin produced in the NAcc mediates depression-like behavior via activating KORs that are local to the NAcc. Dynorphin-positive neurons in the NAcc do project to neighboring neurons in this area, including other dynorphin-positive cells [57] and intra-NAcc administration of a KOR antagonist is sufficient to induce an antidepressant-like effect in the learned helplessness paradigm [26]. Dynorphin-positive cells in the NAcc also project to other brain regions, including the VTA, substantia nigra (SN) and the ventral pallidum [58]. However, while the VTA and SN have relatively high levels of KOR mRNA, their levels of KOR peptide is low and the administration of KOR agonists into the SN and VTA does not induce conditioned place aversion [20] and reduced dopamine levels in the NAcc [59], respectively. Thus, it has been suggested that KORs are produced in the VTA and SN and subsequently transported to the NAcc, where they are expressed on presynaptic terminals [60]–[62]. Therefore, it is likely that down-regulation of prodynorphin in the NAcc induces depressive-like behavior mainly via activation of receptors within the NAcc itself. Future studies will verify this postulation.

In contrast to their role in depressive-like behavior, dynorphin expression in the NAcc does not seem to be involved in anxiety-like behavior. In fact, studies have pointed instead to elements of the amygdala, such as the central nucleus of the amygdala [63] and the basolateral amygdala [30], [31], as regions in which disruption of KOR attenuates anxiety-like behavior, possibly through dysregulation in GABA transmission [63].

Several studies have demonstrated that similarly to the effects of chronic stress [54], repeated exposure to cocaine leads to upregulation of dynorphin in the NAcc [64]. Thus, it has been suggested that dynorphin upregulation mediates the negative emotional effects of cocaine withdrawal, such as hypohedonia [2]. This upregulation may also influence the long-term state resulting from repeated cocaine exposure that is known as “sensitization” and that is thought to contribute to the development of addiction [65]. In rodents, the sensitized state includes increase in the reinforcing and incentive effects of cocaine [66], as well as augmented locomotor response to a cocaine challenge [35]. A large body of evidence suggests that cocaine behavioral sensitization results from increased mesolimbic dopaminergic activity [67]. Given the modulatory effect of KOR activation on dopamine release in the NAcc (see above), the dynorphin/KOR system could play a role in cocaine sensitization. However, manipulations of the dynorphin/KOR system have yielded inconsistent and even paradoxical results. Specifically, systemic co-administration of KOR agonists and cocaine attenuated the development of sensitization to the locomotor stimulatory effect of cocaine [37]–[39]. However, the long-lasting KOR antagonist norBNI also prevented the cocaine locomotor sensitization without affecting the acute response to cocaine [40]. The results of the current study demonstrate that *Pdyn* knockdown in the NAcc did not alter baseline locomotor behavior or the locomotor response to acute cocaine, but inhibited sensitization with repeated administrations. Thus, sensitization developed similarly in shPdyn and shScr rats on the first 4 cocaine treatment days but after three days of abstinence the locomotor response to the cocaine challenge returned to their original levels in the shPdyn rats while remaining heightened in the shScr group.

This pattern of results suggests that dynorphin in the NAcc plays less of a role in the development of cocaine sensitization, but more of a role in supporting the maintenance of the sensitized state. Thus, dynorphin may be particularly essential for the induction of cocaine-induced neuroplastic adaptations in the mesolimbic system that may account for the development of cocaine addiction as a persistent and lasting pathological condition. To this end, one can hypothesize that acute dynorphin-induced suppression of dopamine release decreases the induction of locomotor sensitization but chronic reduction in dynorphin modifies compensatory mechanisms that contribute to expression of sensitization. Alternatively, the fact that both *Pdyn* knockdown and KOR agonists interfere with cocaine sensitization may suggest that specific patterns of dopamine release (or another consequence of dynorphin/KOR activation) determine the long-term effects of cocaine, and that deviation from these patterns, whether an increase or a decrease of activity, will disrupt the development or maintenance of cocaine sensitization.

One limitation of the current study is that the volume of the NAcc in which *Pdyn* was silenced was rather small, due to the limited spread of our AAV2 vectors. It bears noting, however, that this sub region of the nucleus accumbens (dorsal shore of the core and shell) is one of the subregions of the NAcc with the highest immunoreactivity for dynorphin [68]. Nevertheless, although this restricted knockdown was sufficient to induce significant behavioral effects, we cannot rule out that differences between the groups in the elevated plus maze would have emerged had a more extended part of the NAcc been affected by the viral vector. This possibility will be addressed in future studies using a different AAV serotype to increase the transduction volume. In addition, future studies will measure levels of the prodynorphin peptide in order to locate brain regions in which dynorphin release may be reduced following *Pdyn* knockdown in the NAcc. Finally, prodynorphin is the precursor protein for several peptides, including dynorphin A, dynorphin B, and α/β-neo-endorphin [1]. The particular role of each of these in mood regulation and the effects of cocaine still remains to be determined.

In summary, the current study demonstrated that AAV-mediated *Pdyn* knockdown in the NAcc attenuates depressive-like behavior, without affecting anxiety-like behavior, and led to the normalization of the locomotor response to cocaine challenge subsequent to the development of cocaine sensitization. These results support the notion that dynorphin plays an important role in the regulation of mood states and the lasting neuroplastic consequences of repeated cocaine exposure that may be at the core of cocaine addiction.
